# Research on Improving Pretreatment Process of Banana Fiber Fabric with Rare Earth

**DOI:** 10.3390/molecules30234535

**Published:** 2025-11-24

**Authors:** Jie Liu, Wenqi Jiang, Chun Lv, Lingfang Sun, Yongjie Zheng

**Affiliations:** 1 College of Light-Industry and Textile Engineering, Qiqihar University, Qiqihar 161006, China; shearr@126.com; 2 Engineering Research Center for Hemp and Product in Cold Region of Ministry of Education, Qiqihar 161006, China; 3 YUYUE Home Textile Co., Ltd., Binzhou 256623, China; 18204667348@163.com; 4 College of Architecture and Civil Engineering, Qiqihar University, Qiqihar 161006, China; lvc@qqhru.edu.cn

**Keywords:** banana fiber, lanthanum sesquioxide, pretreatment, hydrogen peroxide, process

## Abstract

Scouring and bleaching processes of banana fiber fabric based on rare earth complex Lanthanum sesquioxide (La_2_O_3_) pretreatment were studied. The effects of rare earth content, hydrogen peroxide concentration, sodium hydroxide concentration, temperature, time, and stabilizer concentration on the weight loss ratio, whiteness, capillary effect, and breaking strength of the banana fiber fabric were analyzed. The optimized process was determined by an orthogonal test, namely, the rare earth Lanthanum sesquioxide (La_2_O_3_) 0.25% o.w.f., sodium hydroxide 4.5 g/L, hydrogen peroxide concentration 7.5 g/L, the stabilizer 3 g/L, processed 60 min at 75 °C. Through infrared spectroscopy (IR), Scanning electron microscopy (SEM), X-ray diffraction (XRD), Thermogravimetry (TG), and other analysis, it was proven that the rare earth pretreatment process did not change the molecular structure of the cellulose and had little effect on the fabric thermal stability. The fabric obtained by the rare earth pretreatment process has high whiteness, high breaking strength, less damage to the fiber, good fiber wettability, a high capillary effect, and good handle.

## 1. Introduction

Bananas are grown in about 130 countries and regions around the world. In 2021, the global banana harvest area was stable at about 5.34 million hectares, with an annual production of more than 120 million tons, while the amount of discarded stalks was almost equal to the amount of banana fruits [1]. In the past, the banana stalks could only be used as waste, which could rot for a long time, spread diseases and pests, pollute water sources, and cause environmental deterioration. If these waste banana stalks were developed and applied to textile and other fields, they could not only solve the problem of environmental damage caused by waste stems but also enrich types of natural fibers, bring new industrial chains to the textile industry, and improve the economic benefits of the banana industry, which has good development prospects [2]. Therefore, the development of banana fiber can not only realize the utilization of waste resources and effectively solve the problem of waste banana stems, but also enrich types of new environmentally friendly fibers and realize the rational utilization of resources [3]. Banana fiber is divided into leaf fiber and stem fiber. Compared with banana leaf fiber, banana stem fiber has longer length, better strength and elongation, and more cellulose content, which makes it easy to produce fiber. Therefore, banana stem fibers were selected for textile processing [4,5]. Banana stem fiber is obtained from banana stalks, similar to flax and jute, and its single-fiber length is closer to jute but shorter than flax [6]. The cross section of a banana fiber is round at the waist, with cavities in the middle, and some flat fibers have holes and cracks. The transverse joints of the longitudinal appearance are gently raised, and the shape is flat and straight with cracks. In addition to cellulose, the banana fiber contains hemicellulose, pectin, wax, water-soluble substances, and lignin [7,8]. Although banana fiber is similar to hemp, jute, and flax, it contains a small amount of protein, so banana fibers also have some properties of protein fibers. Its acid resistance is stronger than cotton and hemp fiber but weaker than wool, and its alkali resistance is stronger than wool but weaker than cotton and hemp fiber [9]. Banana fiber (density 1.3610 g/cm^3^) is lighter than flax (density 1.4928 g/cm^3^), and it has good hygroscopicity with about 10% moisture content. The orientation and crystallinity are lower than hemp fiber, but the macromolecular arrangement homogeneity is not as good as that of hemp fiber, and it has better hygroscopicity and dehumidification and lower birefringence than jute and flax [10,11]. Today, banana fiber can be used as a new ecological and environmental protection raw material and has great room for development in aerospace, textile dyeing, and finishing processing [12], as well as medical care and other fields, and it has become a substitute for many non-renewable resources [13,14].

Banana fibers need to be degummed before spinning, with techniques such as physical degumming [15], ultrasonic degumming [16], chemical degumming [17], enzymatic degumming [18,19], and combined degumming [20]. Degummed banana fibers can be blended with other fibers or spun alone [21,22]. The fine processing and production technology of banana fiber completed by Donghua University has passed expert identification, which has reached the international advanced level and is currently used in the production of various exquisite clothing [23]. Fabric made of banana fiber needs to be pretreated before dyeing and finishing, in order to remove impurities in the fiber and the size of the fabric and improve the absorption and whiteness of the fabric.

High-efficiency short-process pretreatment is a process that combines conventional desizing, scouring, and bleaching into one step, which is called the alkali–oxygen one-bath process of desizing, scouring, and bleaching [24]. The decomposition products of hydrogen peroxide (H_2_O_2_) under strong alkaline conditions, such as the perhydroxyl anion, perhydroxyl radical, and hydroxyl radical, can react with the double bond in the coloring matters, destroy the color system, and help to remove impurities [25,26]. This process can improve the processing efficiency and reduce the waste of water and energy. The traditional hydrogen peroxide pretreatment process had a great effect on the fabric breaking strength. To reduce fabric damage caused by pretreatment, some methods such as using an oxygen bleaching activator to reduce the processing temperature were adopted. During our research on using rare earth as an auxiliary to enhance the processing effects, we discovered that rare earth can also improve the effect of the alkali–oxygen one-bath pretreatment for banana fiber fabrics.

Rare earth is the general name for scandium, yttrium, and lanthanide elements, which are located in the third subgroup of the periodic table. Among them, seventeen elements such as neodymium (Nd), cerium (Ce), and lanthanum (La) in the lanthanide series, yttrium (Y), and scandium (Sc) are included [27,28]. Due to the elements’ unique electronic layer structure, rare earth has the advantages of being non-toxic, environmental protection, heat and climate resistance, etc., and is widely used in dyeing and finishing textiles, the organic chemical industry, the mining industry, inorganic chemical industry, electric power, and other fields [29]. An appropriate amount of La_2_O_3_ plays a catalytic role in the alkali–oxygen one-bath pretreatment. Rare earth elements can catalyze the decomposition of H_2_O_2_, selectively generating more hydroxyl radicals with bleaching activity, while inhibiting the excessive production of hydroxyl radicals, which cause fiber damage. This reduces the activation energy of the reaction, enabling the bleaching reaction to proceed efficiently under milder conditions, thereby improving the whiteness while reducing the oxidative damage to cellulose. Meanwhile, rare earth elements can activate the colored substances in the fiber. There is a strong complexation ability between La^3+^ and the coexisting impurities in the fibers [30]. By forming water-soluble complexes, La_2_O_3_ promotes the dissociation of these impurities from the fibers and their dispersion in water, thereby improving the pretreatment effect.

In this paper, the banana fiber fabric was pretreated with rare earth lanthanum sesquioxide (La_2_O_3_), and the banana fiber fabric was desized, scoured, and bleached with hydrogen peroxide in one bath. The experimental results showed that the fabric pretreated with rare earth has high whiteness, little damage, and a good pretreating effect. Therefore, the application of rare earth in the fabric pretreatment process is of great significance.

## 2. Experimental Section

### 2.1. Materials

Banana fiber fabric was obtained from Haikou Experimental Station of the Chinese Academy of Tropical Agricultural Sciences. Lanthanum sesquioxide (La_2_O_3_, purity 99.999%) was purchased from Lianyungang Xinfu Rare Earth Co., Ltd. (Lianyungang, China). Citric acid, H_2_O_2_ (30% *w*/*w*), and NaOH were purchased from Liaoning Quanrui Reagent Co. Ltd. (Jinzhou, China) (analytically pure). The scouring agent DM-1335 and the stabilizer DM-1403 were obtained from Guangdong Demei Fine Chemical Co. Ltd. (Shunde, China) (technical-grade). Penetrant JFC was obtained from Tianjin Kaitong Chemical Reagent Co Ltd. (Tianjin, China) (analytically pure).

### 2.2. Pretreatment Process of Banana Fiber Fabric

The fabric (20 cm × 5 cm) was firstly immersed in a bath which contained [0.1, 0.15, 0.2, 0.25, 0.3, 0.35, 0.4]% o.w.f. La_2_O_3_, pretreated at room temperature for 15 min. The rare earth-pretreated fabric was steeped in a processing bath made up of 2 g/L scouring agent DM-1335, 2 g/L JFC, [0, 1, 2, 3, 4, 5, 6] g/L stabilizer DM-1403, [2, 4, 6, 8, 10, 12, 14] g/L hydrogen peroxide, [2, 4, 6, 8, 10, 12, 14] g/L sodium hydroxide, with processing at [40, 50, 60, 70, 80, 90, 100] °C for [30, 40, 50, 60, 70, 80, 90] min; the bath ratio was 30:1. After being pretreated, these fabrics were then removed and washed in water at 80 °C, 50 °C, and room temperature, respectively, then oven-dried for 3 h until the weight was constant.

The pretreatment process and washing were carried out in regular laboratory glass beakers, with the temperature controlled by the DK-98-1 electronic constant temperature water bath pot (Beijing Yongguangming Medical Instrument Factory, Beijing, China). After pretreatment, the fabrics were successively washed at 80 °C for 10 min, 50 °C for 5 min, and room temperature for 5 min. Then, the washed fabrics were placed in a 202-AOS electrothermal constant temperature drying oven (Tianjin Huabei Experimental Instrument Co., Ltd., Tianjin, China) and dried at 105 °C for 3 h, until reaching a constant weight.

### 2.3. Performance Test

The whiteness of banana fiber fabric was measured with YQ-Z-48A whiteness chromograph (Hangzhou Qingtong Boke Automation Technology Co., Ltd., Hangzhou, China). According to GB/T 17644-2008 (Textile Fiber Whiteness Chroma Test Method) [31], the sample was folded into four layers (the fabric was flat, and the surface grain direction was as consistent as possible) and placed on the tester tray. The whiteness value of the sample is the average of the test results in five different directions.

The breaking strength of the fabric was tested using YG (B) 026 D-250 electronic fabric strength tester (Wuhan Guoliang Instrument Co., Ltd., Wuhan, China). According to GB/T3923.1-2013 (Textiles—Tensile properties of fabrics—Part 1: Determination of maximum force and elongation at maximum force using the strip method) [32], the sample to be measured was clamped, we pressed the switch, the sample continued to stretch; we measured it 5 times then took the average value.

The weight loss ratio was calculated by Formula (1), using the oven process.

Weight loss ratio (%) = [(*W*_1_ − *W*_2_)/*W*_1_] × 100% (1)

where *W*_1_ and *W*_2_ are the dry weight of gray cloth and the treated fabric, respectively.

The fabric capillary effect was measured with the YG(B)871 capillary effect tester (Wenzhou Darong Textile Standard Instrument Factory, Wenzhou, China). Referring to FZ/T 01071-2008 (Textiles—Test method for capillary effect) [33], the fabric upper end was held on the tester stand, we held the bottom end of the fabric with a small clip, these clips were thoroughly immersed in water, then we began to measure, recorded the water level height at 30 min, and calculated the average of the five test results.

### 2.4. Hydrogen Peroxide Decomposition Ratio Measurement

The prepared 2 mL of bleaching solution was pipetted into a conical bottle filled with water and 6 mol/L sulfuric acid solution and then titrated with the pre-prepared potassium permanganate (KMnO_4_) standard solution (0.02 mol/L). When the solution changes from colorless to red (the red color does not disappear within 30 s), we recorded the volume of potassium permanganate solution consumed, repeated it twice, calculated the average value, then calculated the hydrogen peroxide decomposition ratio using formula (2).

Hydrogen peroxide decomposition ratio (%) = [(Vo − Vt)/Vo](2)

where Vo is the volume of KMnO_4_ standard solution consumed by the bleaching bath; Vt is the volume of KMnO_4_ standard solution consumed by the residual bleaching bath at time t.

### 2.5. Characterization

SEM (scanning electron microscopy) (S-3400, Hitachi, Japan) was used to characterize the microtopography of the banana fiber fabric before and after rare earth treatment. The samples being analyzed were sprayed with gold to improve the electrical conductivity. The X-Ray diffraction (XRD) instrument (Rigaku SmartLab SE, Hitachi, Japan) was employed to collect 2000 counts in the range of 5–45° with a step size of 0.02° and 2°/min scanning rate to study the crystal structure of powders produced from banana fiber fabric. Note that the metallic background was pre-deducted and the diffraction patterns obtained were not further smoothed. An XPS photoelectron spectrometer (ESCALAB250Xi, Thermo Fisher Scientific, Bend, OR, USA) was used to analyze the surface element content of the banana fiber. The FTIR spectrum was measured by using Spectrum100 FTIR spectrometer (PE, Cincinnati, OH, USA) in the scanning region of 4000~500 cm^−1^ and with 32 scanning times per wavelength. The fabrics before and after treatment were mixed with KBr, respectively, then pressed into thin slices to be measured. The fabric before and after pretreatment was shorn into a very fine powder. The thermal property of the banana fiber was analyzed with a synchronous thermal analyzer (STA 449 F3 Jupiter, NETZSCH, Selb, Germany).

## 3. Results and Discussion

### 3.1. Factors Affecting the Pretreatment Performance of Banana Fiber Fabric

#### 3.1.1. Rare Earth La_2_O_3_ Concentration

**Figure 1:**
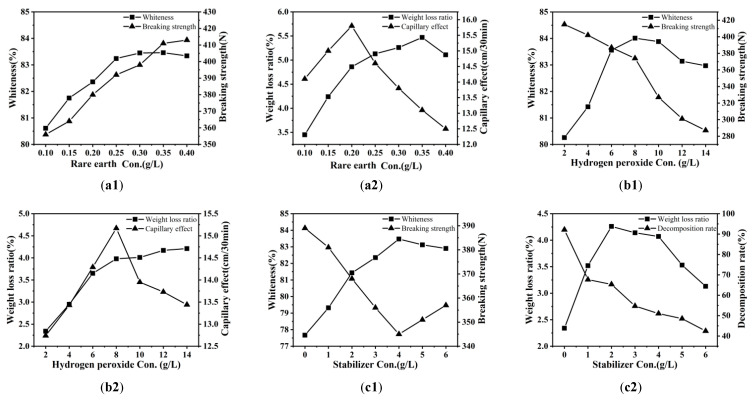

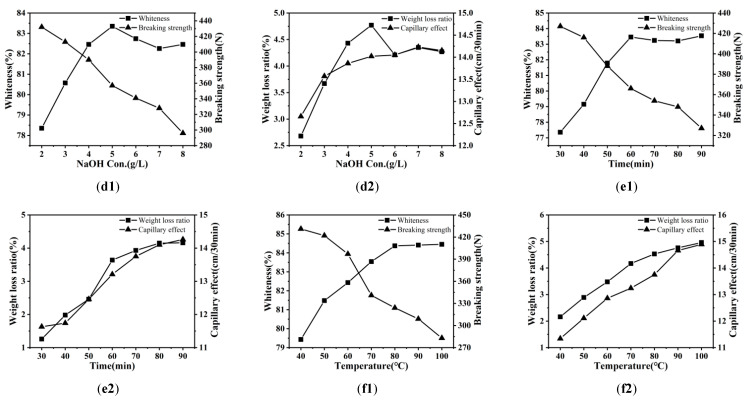
Factors affecting the pretreatment performance of banana fiber fabric: (**a1**,**a2**) rare earth La_2_O_3_ concentration; (**b1**,**b2**) hydrogen peroxide concentration; (**c1**,**c2**) stabilizer concentration; (**d1**,**d2**) NaOH concentration; (**e1**,**e2**) temperature; (**f1**,**f2**) time.

Rare earth can activate colored substances in the banana fiber, make it easier for it to react with the bleaching agent hydrogen peroxide, and reduce the activation energy of the bleaching reaction in the pretreatment; that is, the addition of rare earth has a catalytic function in banana fiber bleaching [34]. Figure 1(a1,a2) shows the influence of rare earth La_2_O_3_ consumption on the breaking strength, whiteness, capillary effect, and weight loss ratio of banana fiber fabric pretreated for 60 min at 80 °C using 0.10, 0.15, 0.20, 0.25, 0.30, 0.35, 0.40% o.w.f. La_2_O_3,_ 10 g/L H_2_O_2_, 5 g/L NaOH, and 3 g/L stabilizer.

In Figure 1(a1,a2), with the increase in rare earth consumption, the rare earth ion, as the center ion, had a strong complexation with the ligand containing lone-pair electron groups, entered the amorphous region of the fiber, and formed a complex employing the bonding of the coordination bond and the covalent bond, acting as a crosslinking agent, thus improving the strength of the fabric. Rare earth ions can also swell the fiber and relax the structure; thus, the rare earth elements can form complexes with decomposed impurities containing N, O, S, and other elements. After washing, they were dispersed in the solution, which helped hydrogen peroxide to decompose effectively and destroyed the color base group in the fiber, improving both the capillary effect and whiteness. However, when the rare earth La_2_O_3_ concentration reached 0.3% o.w.f., the whiteness of the fabric decreased slightly. This is because the rare earth concentration was too high, and the formed complex produced precipitates, resulting in an unstable treatment liquid, affecting the pretreatment effect, and reducing the weight loss ratio and capillary effect. After comprehensive consideration, the concentration of rare earth elements was selected as 0.3%o.w.f.

#### 3.1.2. Hydrogen Peroxide Concentration

Hydrogen peroxide used as a bleaching agent can destroy the conjugated system of color matters through an oxidation reaction to achieve the purpose of improving whiteness. Figure 1(b1,b2) shows the influence of H_2_O_2_ con. on the four processing performances of the banana fiber fabric treated for 60 min at 80 °C using 0.3% o.w.f. rare earth La_2_O_3_, 3 g/L stabilizer, 2, 4, 6, 8, 10, 12, 14 g/L hydrogen peroxide, and 5 g/L NaOH. As seen in Figure 1(b1,b2), with the increase in H_2_O_2_ con., the capillary effect, whiteness, and weight loss ratio gradually increased, but the breaking strength decreased gradually. When the hydrogen peroxide concentration was about 8 g/L, the whiteness and capillary effect were the best; when the hydrogen peroxide concentration was higher than 8 g/L, the whiteness and capillary effect gradually decreased. When the concentration of hydrogen peroxide is lower, the amount of active components produced by H_2_O_2_ decomposition is insufficient, making them unable to effectively destroy impurities and color matters, resulting in an unsatisfactory treatment effect. As the concentration of H_2_O_2_ increases, under alkaline conditions, more hydrogen peroxide molecules are activated, and through reactions, high-reactive hydroxyl radicals ·OH and peroxide radicals HOO· are generated. These strong oxidizing free radicals can break the macromolecular chain through oxidation, causing the degradation of the molecular chains of natural impurities, thereby making them easier to detach from the fibers, as well as attacking and destroying the structure of the conjugated coloration groups, achieving decolorization and bleaching. With the removal of these degradation products during the washing process, the cleanliness and hydrophilicity of the fiber surface are improved, and macroscopically, the capillary effect and whiteness increase are manifested. However, an excessive concentration of hydrogen peroxide caused the molecular chain of the fiber to be oxidized and destroyed, and the secondary hydroxyls of C_2_ and C_3_ in the glucose molecules were oxidized to the keto group and diketone group, the macromolecular chain was broken, the degree of polymerization was reduced, and the breaking strength decreased gradually. The continuous increase in fabric weight loss ratio did not completely mean that the pretreatment effect must be good; it may also be caused by excessive fiber damage. Therefore, combined with whiteness and the degree of fiber damage, the H_2_O_2_ con. should be 8 g/L.

#### 3.1.3. Stabilizer Concentration

The presence of heavy metals such as iron and copper in the bleaching bath will cause the hydrogen peroxide to decompose too quickly, resulting in the loss of active ingredients in bleaching and damaging fabrics. Therefore, oxygen bleaching stabilizer is needed in the bleaching process; its function is to bind heavy metal ions and prevent their catalytic effect on hydrogen peroxide decomposition, to achieve the expected treatment effect without excessive fiber damage. Figure 1(c1,c2) shows the effects of stabilizer concentration on breaking strength, whiteness, weight loss ratio, and the hydrogen peroxide decomposition ratio of the banana fiber fabric treated for 60 min at 80 °C using 0.3% o.w.f. rare earth La_2_O_3_, 8 g/L hydrogen peroxide, 0, 1, 2, 3, 4, 5, 6 g/L stabilizer, and 5 g/L NaOH. In Figure 1(c1,c2), when the stabilizer concentration increased up to 4 g/L, the fabric whiteness increased from 77.67% to 83.47%, indicating that an appropriate amount of stabilizer can control the hydrogen peroxide decomposition ratio, inhibit the rapid generation of hydrogen peroxide free radicals, improve the whiteness of the fabric, reduce the oxidation of cellulose, and reduce the damage to the fabric. After the stabilizer concentration increased again, the whiteness of the fabric began to decrease. The reason was that with the stabilizer concentration increasing again, the stabilizer over-inhibited H_2_O_2_ decomposition, enabling the removal of those impurities; the weight loss ratio decreased, but the fabric damage became smaller and the breaking strength increased. To achieve both whiteness and breaking strength, the stabilizer concentration was selected to be about 3 g/L.

#### 3.1.4. Sodium Hydroxide Concentration

The presence of sodium hydroxide (NaOH) can adjust the pH value of the bleaching bath and promote the stable decomposition of H_2_O_2_ to produce more active ingredients so that the oxidation capacity of the hydrogen peroxide is high for a certain period, which is conducive to the removal of impurities in the fiber. Figure 1(d1,d2) shows the effects of NaOH con. on the four processing performances of the banana fiber fabric treated for 60 min at 80 °C using 0.3% o.w.f. rare earth La_2_O_3_, 8 g/L H_2_O_2_, 3 g/L stabilizer, 2, 3, 4, 5, 6, 7, 8 g/L NaOH. In Figure 1(d1), with NaOH con. increasing, the fabric whiteness enhanced but the breaking strength weakened. After that, the fabric whiteness decreased but the breaking strength rapidly decreased with the increase in NaOH con. The reason was that too high a concentration of NaOH caused the hydrogen peroxide to undergo violent decomposition; the generated hydroxyl free radicals and perhydroxyl free radicals not only oxidized the impurities but also oxidized the cellulose of the fiber, so that the fabric was damaged, which affected the pretreatment effect. The higher the NaOH con., the more obvious the influence on the capillary effect and weight loss ratio of the banana fiber fabric (Figure 1(d2)), but the increase in weight loss ratio may also be caused by fiber damage. To avoid excessive damage to fibers, the concentration of NaOH was about 5 g/L.

#### 3.1.5. Processing Time

Processing time is also an important factor that influences the effect of fabric processing. Figure 1(e1,e2) shows the effects of time on the four processing performances of the banana fiber fabric treated using 0.3% o.w.f. La_2_O_3_, 8 g/L H_2_O_2_, 3 g/L stabilizer, and 5 g/L NaOH at 80 °C for 30, 40, 50, 60, 70, 80, and 90 min, respectively. As seen in Figure 1(e1,e2), the weight loss ratio, whiteness, and capillary effect of the fabric increased gradually with the extension of bleaching time, but the breaking strength showed a downward trend. If the processing time was not too long, the reaction was incomplete between H_2_O_2_ and impurities; namely, the impurities could not be destroyed, and the processing effect could not be achieved. With the time extension, hydrogen peroxide gradually decomposed and acted on the fabric and the size of the warp yarns, the impurities of the fabric were removed gradually, the fabric hygroscopic property was enhanced, the capillary effect value was increased, and whiteness was improved. Too long a bleaching time intensified the oxidation of cellulose by hydrogen peroxide, resulting in a continuous decrease in breaking strength, causing fabric damage, while the whiteness was not significantly improved. At the same time, it also increased energy consumption and production costs. After a comprehensive analysis, the bleaching time was about 60 min.

#### 3.1.6. Processing Temperature

Figure 1(f1,f2) shows the influence of temperature on the four processing performances of the banana fiber fabric treated for 60 min using 0.3% o.w.f. La_2_O_3_, 8 g/L H_2_O_2_, 5 g/L NaOH, and 3 g/L stabilizer at 40, 50, 60, 70, 80, 90, and 100 °C. As seen in Figure 1(f1,f2), when the temperature increased from 40 °C to 80 °C, the capillary effect, whiteness, and weight loss ratio were significantly improved as the temperature rose, while the breaking strength was significantly decreased. The reason was that the increase in temperature increased the hydrogen peroxide decomposition ratio and the sizes of the warp yarns, and the impurities of the fabrics were more thoroughly removed, so that the fabric hygroscopicity improved and the capillary effect and weight loss ratio were enhanced constantly. At the same time, the rapid ionization of hydrogen peroxide produced effective strong oxidizing substances that destroyed the color matter system in the fiber, resulting in the whiteness of the fabric increasing. When the temperature was higher than 80 °C, the whiteness, capillary effect, and weight loss ratio did not enhance significantly with temperature rises, and the strength continued to decrease. The reason was that when H_2_O_2_ con. and the processing time were maintained, the hydrogen peroxide decomposition consumption ratio was proportional to the temperature, and an appropriate high temperature was conducive to hydrogen peroxide decomposition. However, if the temperature was too high, hydrogen peroxide decomposed too quickly, and the active ingredients were released too fast, easily escaped into the air, and could not be applied to the fabric, so the fabric would be more damaged at high temperatures. The bleaching temperature should be about 80 °C.

### 3.2. Orthogonal Optimization of the Banana Fiber Fabric Pretreatment Process

The pretreatment process was optimized by an L_9_ (3^4^) orthogonal test. The orthogonal test results and range analysis are shown in Table 1.

As can be seen in Table 1, the order of the influencing factors for whiteness was A > B > C > D, the rare earth concentration had the maximum impact on whiteness, and the optimal process scheme was A_2_B_2_C_2_D_2_, while the order for the breaking strength was B > A > D > C, the hydrogen peroxide concentration was the most influential factor of the fabric breaking strength, and the optimal scheme was A_2_B_3_C_1_D_2_. Considering the influence of whiteness and breaking strength on subsequent processing and the importance of influencing performance indexes, the optimal scheme A_2_B_2_C_1_D_2_ was finally determined to avoid excessive damage to banana fiber during processing and meet the requirements of subsequent printing and dyeing regarding whiteness. So the optimized process parameters of the banana fiber fabric pretreatment process were as follows: 0.25% o.w.f. rare earth, 7.5 g/L H_2_O_2_, 3 g/L stabilizer, and 4.5 g/L NaOH, processed for 60 min at 75 °C. Validation experiments were carried out using the optimized process parameters of the above orthogonal experiment. The performance results showed that the fabric properties were a whiteness of 84.16%, breaking strength of 416 N, and capillary effect of 14.26 cm/30 min, which were superior to those of all preceding experiments. The optimum process was reasonable and feasible.

### 3.3. Comparison of Properties Between Rare Earth La_2_O_3_/H_2_O_2_ Pretreatment and Traditional Pretreatment of Banana Fiber Fabric

#### 3.3.1. Whiteness, Breaking Strength, and Capillary Effect of the Fabric

The properties of banana fiber fabric treated by optimized rare earth pretreatment process were tested and compared with those treated by the traditional process. The properties of fabrics treated by different methods are compared in Table 2. It is obvious that the fabric whiteness, capillary effect, and breaking strength treated with rare earth were better than the corresponding indexes of the fabric treated with the traditional process. The higher whiteness and capillary effect indicated that the removal of the sizes and impurities was more thorough than those of traditional processing, and the pretreatment of rare earth was the most important. Although the fabric breaking strength decreased by 37.80 N compared with the gray fabric, it was significantly less than the 77.80 N decrease in the traditional process, indicating that the rare earth pretreatment process caused much less damage to the fiber than the traditional process.

#### 3.3.2. Scanning Electron Microscopy (SEM)

**Figure 2:**
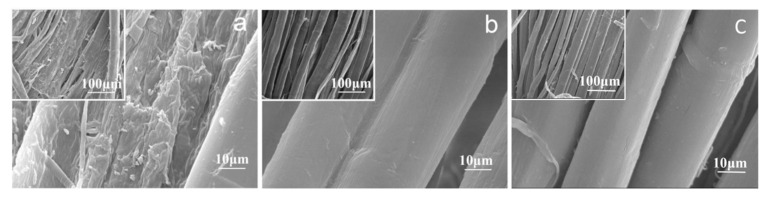
SEM images of the banana fiber treated with various methods: (**a**) unprocessed; (**b**) rare earth La_2_O_3_/H_2_O_2_ process; (**c**) traditional H_2_O_2_ process.

The banana fiber fabrics treated by different methods were examined by using the Hitachi S-3400 scanning electron microscope with 400× and 3000× scanning values. The SEM images are shown in Figure 2. It shows that the banana fiber surface changed obviously after pretreatment. Through pretreatment, the banana fiber surface became clean, flat, and smooth, but the flatness of the banana fiber surface processed by different methods was also different. There was a lot of granular solid matter on the untreated banana fiber surface (Figure 2a), which were the warp size and the impurities. The fiber surface pretreated with rare earth La_2_O_3_/H_2_O_2_ was clean, flat, and smooth, indicating that the sizes and impurities had been fully removed, and the fiber was vertically straight, with concave and convex transverse joints similar to the shape of wool scales, and the deep grooving can be clearly seen throughout the fiber surface, that was the reason for good moisture absorption of the banana fiber, and the fiber was damaged but not serious (Figure 2b). The fiber surface treated by the traditional H_2_O_2_ process was also relatively clean and smooth, but the degree of cleanliness and smoothness was still not as good as that of the rare earth La_2_O_3_/H_2_O_2_ treatment, and the damage to the fiber was greater (Figure 2c), indicating that the rare earth La_2_O_3_/H_2_O_2_ pretreatment process was superior to the traditional process.

#### 3.3.3. XRD

**Figure 3:**
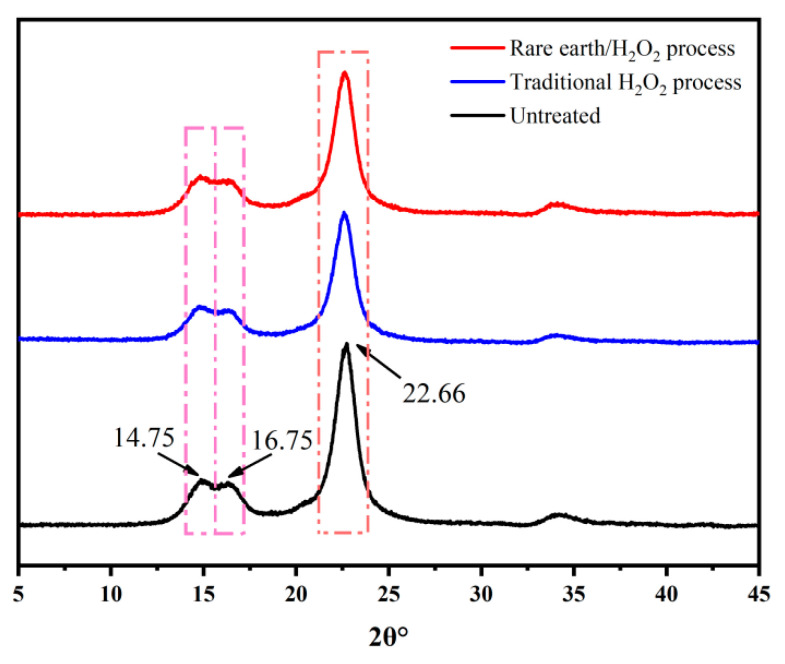
X-ray diffraction patterns of banana fiber fabric.

The XRD pattern of banana fiber fabric is shown in Figure 3. It is obvious that the XRD peaks and curves of the banana fiber fabric treated by different methods are similar to those of unprocessed fabrics. The banana fiber cellulose crystalline structure conforms to cellulose I; namely, the diffraction peaks (14.75°, 16.75°, and 22.66°) match with the diffraction planes (1–10), (110), and (200), respectively [35,36]. It shows that the crystalline structure of the fiber has not been changed by pretreatment; that is, the pretreatment did not damage the fiber structure. The difference between samples is that the X-ray diffraction peak intensity of the treated fabric has decreased. The reason may be that fibers have been damaged during the pretreatment process; the fiber has been damaged by oxidation, resulting in a decrease in crystallinity. However, due to the pretreatment of the fabric with the rare earth complex in advance, rare earth enters the amorphous zone of the fiber and forms a complex through the bonding of the coordination bond and the covalent bond, which plays a certain crosslinking role. Therefore, the decrease in the degree of the fiber crystal in rare earth La_2_O_3_/H_2_O_2_ treatment is smaller than that in the traditional process, and the fiber damage is smaller. This meant that the natural crystal distribution of banana fiber was maintained after pretreatment, and the cellulose structure was not changed after pretreatment.

#### 3.3.4. XPS

**Figure 4:**
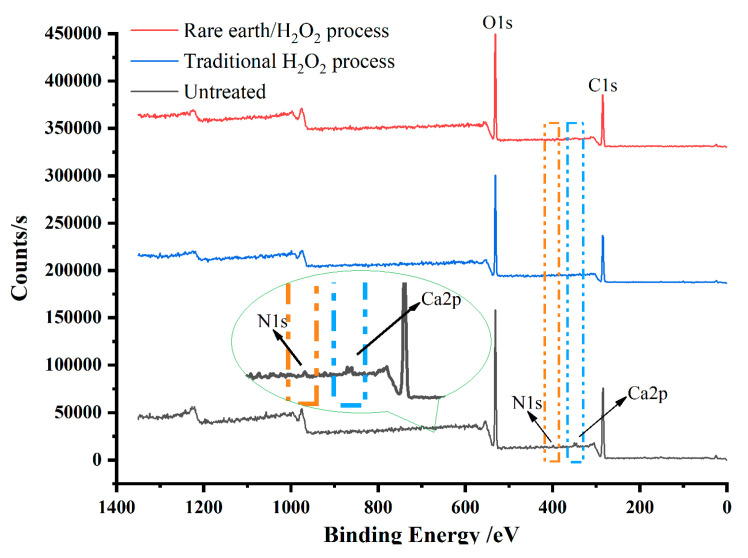
X-ray photoelectron spectroscopy of surface elements of banana fiber fabric.

XPS tests were carried out on raw fabric and the pretreated fabric to observe the changes in the elements present on the fabric surface. The element content of the banana fiber surface is seen in Figure 4 and Table 3. It is obvious that the binding energies of 284.37 eV, 349.9 eV, 398.8 eV, and 531.07 eV are the characteristic absorption peaks of C1s, Ca2p, N1s, and O1s, respectively. Among them, the binding energy 284.37 eV corresponds to C-C, and the binding energy 398.8 eV corresponds to C=N. After pretreatment, the characteristic absorption peaks of N1s and Ca2p disappeared, the content of carbon on the fiber decreased, the content of oxygen increased, and the content of nitrogen and calcium decreased to zero. This is because that C=N bond was attacked by the perhydroxyl anion, so that the sizes and impurities of the fabric were thoroughly removed, resulting in a decrease in the content of carbon, nitrogen, and calcium on the fabric surface and an increase in oxygen content, which was also the reason for the increase in fabric whiteness. Compared with the traditional H_2_O_2_ pretreatment process, the fiber treated by the rare earth La_2_O_3_/H_2_O_2_ pretreatment process had low carbon content and high oxygen content, indicating that the rare earth La_2_O_3_/H_2_O_2_ treatment removed impurities more thoroughly, and the process was more effective.

#### 3.3.5. Infrared Spectrogram (FT-IR)

**Figure 5:**
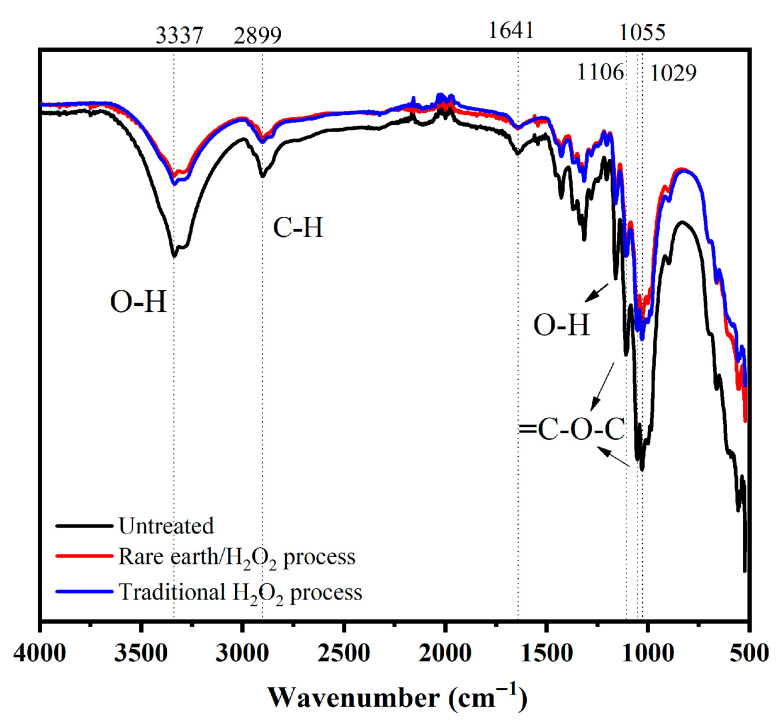
FT-IR spectrum of banana fiber fabric.

The infrared spectrum of the treated banana fiber fabric was measured and compared with that of the gray fabric. The infrared spectrum of the samples is shown in Figure 5. The peak at 3334 cm^−1^ is the O-H stretching vibration absorption peak of cellulose and hemicellulose macromolecules in the molecular structure of banana fiber, and the peak at 2899 cm^−1^ is due to the stretching vibration absorption peak of the saturated C-H bond, such as -CH_3_ and -CH_2_-; both of these are characteristic absorption peaks for cellulose. The stretching vibration absorption peak at 1641 cm^−1^ represents the skeleton structure of the benzene ring and is the lignin characteristic absorption peak. At 1055 cm^−1^ and 1106 cm^−1^, the peaks were C-O stretching vibration absorption peaks of C_2_, C_3_, and C_6_ secondary alcohols and primary alcohols of cellulose macromolecules, respectively. At 1029 cm^−1^, the stretching vibration absorption peaks represent C-O-C and β-1, 4-glucoside bonds between cellulose and hemicellulose. By comparing the three infrared spectral curves in Figure 5, it can be found that the pretreatment of the banana fiber fabric did not change the structure of cellulose.

#### 3.3.6. Thermal Gravimetric Analysis

**Figure 6:**
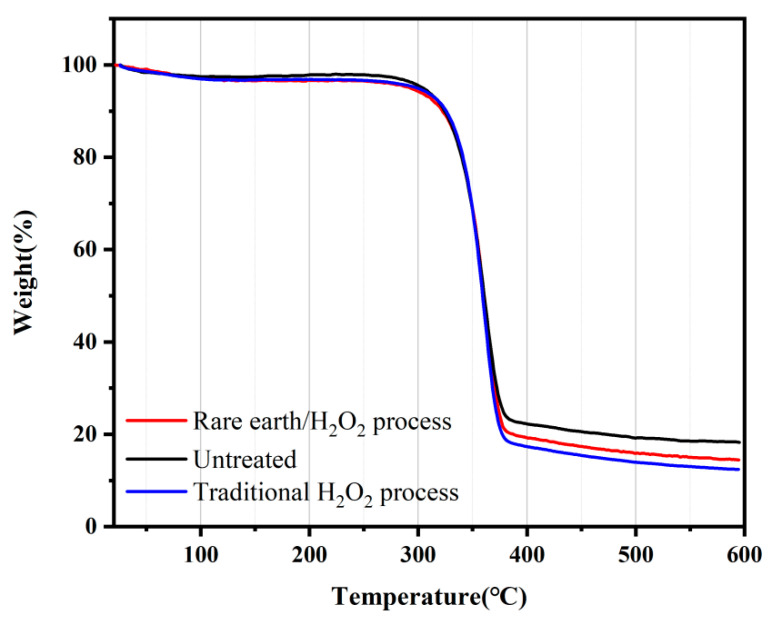
Thermal gravimetric analysis curve of the banana fiber fabric.

To analyze the effect of pretreatment on fabric thermal stability, the thermal performance index of the pretreated and untreated banana fiber fabric was tested. The change in the fabric weight with temperature before or after treatment is seen in Figure 6. The initial cracking temperature of the untreated banana fiber fabric was 305 °C. When the temperature increased to 336.27–385.27 °C, the weight of the banana fiber fabric rapidly decreased, 385.27 °C was the maximum cracking temperature, and the fabric residue ratio was 18.25% at 600 °C. The initial temperature and maximum cracking temperature of the banana fiber fabric treated with rare earth La_2_O_3_/H_2_O_2_ were 321 °C and 377.66 °C, respectively, and the residue ratio was 14.45% at 600 °C. The initial temperature and maximum cracking temperature of the banana fiber fabric treated with traditional hydrogen peroxide were 308 °C and 379.51 °C, respectively, and the residue ratio at 600 °C was 12.42%. The thermogravimetric curves of banana fiber fabrics showed the same trend before and after treatment. When the initial cracking temperature was reached, the fabric weight decreased rapidly; once the temperature rose above the maximum cracking temperature, the fabric weight decreased slowly and tended to be stable. The hydroxyl groups at positions 2 and 3 of the glucose rings of cellulose macromolecules were oxidized to aldehyde groups during the pretreatment process, resulting in the breakage of part of the glucose rings of the fiber and the destruction of hydrogen bonds in the crystallization zone of the fiber. After the pretreatment, the covalent bonding ability between molecules in the fiber became worse, and the molecular crystallinity decreased. At this stage, the weight of the fabric decreased significantly. From the beginning of carbonization to the end of carbonization, the fiber emitted reaction heat, and the glucoside bond in the cellulose structure was broken and decomposed into volatile compounds such as CO_2_ and CH_4_ and tar products [37]. As the temperature continued to rise, the fiber entered the stage of uniform weight loss, and the decline rate gradually slowed down. In general, the thermal weight of banana fiber fabrics did not change much before and after treatment, indicating that pretreatment had little effect on the fabric thermal stability, and rare earth La_2_O_3_/H_2_O_2_ treatment had less effect on the fabric than traditional H_2_O_2_ treatment.

## 4. Conclusions

The optimal pretreatment process of the banana fiber fabric with rare earth La_2_O_3_/H_2_O_2_ was determined: the banana fiber fabric was pretreated for 15 min in a rare earth La_2_O_3_ pretreatment solution of 0.25% o.w.f., then, the pretreated fabric was processed for 60 min at 75 °C in a treatment bath of H_2_O_2_ (30%) 7.5 g/L, NaOH 4.5 g/L, stabilizer DM-1403 3 g/L, refining agent DM-1335 2 g/L, and JFC 2 g/L, and the bath ratio was 30:1. The SEM and other performance test results showed that compared with the traditional pretreatment process of hydrogen peroxide, rare earth La_2_O_3_/H_2_O_2_ pretreatment made the banana fiber fabric show higher whiteness and capillary effects and better effects of desizing and impurity removal, and the process conditions were milder, the decrease in breaking strength of the fabric was obviously reduced, and the damage caused by the traditional pretreatment was reduced. The results of XRD and IR spectra showed that the pretreatment process did not change the molecular structure of cellulose and maintained the natural crystal distribution. The XPS test results showed that the characteristic peaks of N1s and Ca2p disappeared, the carbon content decreased, and the oxygen content increased in the treated banana fiber. Thermal gravimetric analysis (TG) showed that pretreatment had little effect on the banana fiber fabric thermal stability.

## Figures and Tables

**Table 1:** Orthogonal test and results.

No	A (La_2_O_3_ Con.) %	B (H_2_O_2_ Con.) g/L	C (NaOH Con.) g/L	D (Temperature) °C	Whiteness %	Breaking Strength N
1	0.20	7.0	4.5	65	82.30	368.60
2	0.20	7.5	5.0	75	82.87	386.00
3	0.20	8.0	5.5	85	82.81	375.40
4	0.20	8.5	6.0	95	82.97	368.00
5	0.25	7.0	5.0	85	83.71	367.40
6	0.25	7.5	4.5	95	83.89	387.20
7	0.25	8.0	6.0	65	82.51	413.00
8	0.25	8.5	5.5	75	83.53	400.40
9	0.30	7.0	5.5	95	81.85	371.40
10	0.30	7.5	6.0	85	82.73	376.20
11	0.30	8.0	4.5	75	82.87	418.20
12	0.30	8.5	5.0	65	83.25	375.40
13	0.35	7.0	6.0	75	83.03	354.80
14	0.35	7.5	5.5	65	83.56	397.00
15	0.35	8.0	5.0	95	83.05	369.40
16	0.35	8.5	4.5	85	82.86	387.60
K_1_	82.74	82.72	82.98	82.91		
K_2_	83.41	83.26	83.22	83.08		
K_3_	82.68	82.81	82.94	83.03		
K_4_	83.13	83.15	82.81	82.94		
R	0.73	0.54	0.41	0.17		
K_1_^′^	374.50	365.60	390.40	388.50		
K_2_^′^	392.00	386.60	374.50	389.80		
K_3_^′^	385.30	394.00	386.00	376.60		
K_4_^′^	377.20	382.90	378.00	374.00		
R’	17.50	28.40	15.60	15.80		

**Table 2:** Comparison of properties of fabrics treated by different methods.

Processing Method	Whiteness (%)	Breaking Strength (N)	Capillary Effect (cm/30 min)
Untreated	68.35 ± 0.05	453.80 ± 4.56	10.35 ± 0.02
Traditional H_2_O_2_ process	82.66 ± 0.14	376.00 ± 3.76	14.08 ± 0.01
Rare earth La_2_O_3_/H_2_O_2_ process	84.25 ± 0.07	416.00 ± 5.20	14.26 ± 0.02

**Table 3:** The surface element content of banana fiber fabric.

Processing Method	Surface Element Content (%)			
	C1s	O1s	N1s	Ca2p
Untreated	63.53	33.19	2.21	1.07
Traditional H_2_O_2_ process	61.98	38.02	0	0
Rare earth La_2_O_3_/H_2_O_2_ process	60.21	39.79	0	0

## Data Availability

All data is included in the manuscript in the form of figures, graphics, and tables.
